# First description of the male with redescription of the female of *Araneus strandiellus* Charitonov, 1951 (Araneae, Araneidae)

**DOI:** 10.3897/zookeys.205.3077

**Published:** 2012-07-04

**Authors:** Yuri M. Marusik, Anna Šestáková, Mikhail M. Omelko

**Affiliations:** 1 Institute for Biological Problems of the North of the Russian Academy of Sciences, Portovaya Str. 18, 685000 Magadan, Russia; 2 Zoological Museum, University of Turku, FI-20014, Turku, Finland; 3 Department of Zoology, Faculty of Natural Sciences, Comenius University, Mlynská dolina, 84215 Bratislava, Slovakia; 4 Far Eastern Federal University, Sukhanova, 8, Vladivostok 690950 Russia; 5 Gornotaezhnaya Station FEB RAS, Gornotaezhnoe Vil.,Ussuriyski Dist., Primorski Krai 692533 Russia

**Keywords:** Central Asia, orb-weaver, taxonomy, redescription

## Abstract

Redescription of Central Asian orb-weaver *Araneus strandiellus* Charitonov, 1951, only known from the original description of female. The male of this species, previously unknown, is described here for the first time.

## Introduction

Araneidae with 3029 species belonging to 168 genera is third largest spider family (Platnick 2012). The most species rich genus in the family and possibly in the whole order is *Araneus* Clerck, 1757. It encompasses 668 species distributed throughout the globe (Platnick 2012). The genus is studied unevenly in different parts of the world. The most comprehensive studies were made in the Nearctic (Levi 1971, 1973), Europe (Grasshoff 1968; Šestáková et al. 2009), China (Yin et al. 1997) and Japan (Tanikawa 2007, 2009). Only few attempts were made to split the genus into smaller and more natural groups. The most significant contribution was made by Archer (1951a-c, 1958) who described or revalidated over a dozen of genera and subgenera. Most of these taxa were synonymized with *Araneus* by Levi (1971, 1973).

The orb weaving spider *Araneus strandiellus* Charitonov, 1951 is only known by female and since the description was never considered in any other taxonomical publication (cf. Platnick 2012). This species was originally described from northern Tajikistan on the basis of the female holotype. Besides the type locality, the species has been reported from Uzbekistan (Marusik 1989), western Kazakhstan (Pavlenko 1985) and Tuva in South Siberia (Logunov et al. 1998; Marusik et al. 2000). In the original description Charitonov (1951) defined main features of the new species in detail, but did not compare it with any other *Araneus*.

While working with Araneidae material from Siberia and Central Asia we found several samples of *Araneus strandiellus* containing both sexes. The main goal of this paper is description of the male for the first time, and providing detailed redescription of the female.

## Material and methods

Microphotographs were made with an Olympus Camedia E-520 camera attached to an Olympus SZX16 stereomicroscope at the Zoological Museum, University of Turku. Digital images were montaged using “CombineZP” image stacking software. Figures were edited in Corel Photo Paint X4 and Corel Paint Shop Pro Photo X2. Specimens where photographed while placed in dish with paraffin on the bottom filled with 70% ethanol and using different sized holes to keep the samples in the required position. Studied material is deposited in Department of Zoology, Perm State University (PSU), Zoological Museum of Moscow State University (ZMMU), Siberian Zoological Museum of the Institute for Ecology and Systematics of Animals (ISEA), Institute for Biological Problems of the North, Magadan (IBPN) or Alexander V. Gromov (Almaty, Kazakhstan) personal collection (AGA). All measurements are in millimetres.

## Taxonomy

### Araneus strandiellus

Charitonov, 1951

http://species-id.net/wiki/Araneus_strandiellus

Figs 1 –14, 18 –21

Araneus strandiellus Charitonov 1951: 210, f. 2a-b (♀).
Araneus strandiellus : Pavlenko 1985: 153; Marusik 1989: 41; Marusik et al. 1990: 17; Mikhailov 1997: 116; *Araneus strandiellus*: Logunov et al. 1998: 130; Marusik et al. 2000: 13.

#### Material examined.

Holotype ♀ (PSU), **TAJIKISTAN**, Varzob botanical Station, 30.07.1945 (V.V.Gussakovski) [ca 38°50'N, 68°50'E]. **KAZAKHSTAN**, ***Almaty*** Area: 1♂ 3♀ 1juv. (ZMMU), environs of Bakans Town, tugai and thicket, June 1986 (Ch.K. Tarabaev); 1♀ (AGA), Charyn River canyon, Sartogai Boundary, 12.06.1993 (S.V. Ovchinnikov). **RUSSIA, *Tuva***: 1♂ 4♀ (IBPN), Tere-Khol’ Lake, Sharlaa Stand and around, 40°01.47'N, 95°03.45'E, 1150 m, 6–14.07.1996 (Yu.M.Marusik); 1♂ 1♀ (ISEA), Tere-Khol’ Lake, SE shore, Eder-Elezin Sands (Desert), 1150 m, 12.07.1993 (Yu.M. Marusik); 1♂ (ISEA), Tere-Khol’ Lake, S shore, sands with sparse *Caragana* shrubs, 6–26.05.1990 (O. Lyakhov).

#### Diagnosis.

Habitus, pattern and copulatory organs of *Araneus strandiellus* resemble only those in *Araneus pallasi*. Both species have simple, weakly sclerotised epigyne with inflexible scapus; males lack stipes, subterminal apophysis, embolic cup, and have weakly sclerotised conductor; long filamentous embolus; long (as embolus), narrow terminal apophysis; median apophysis with one prolaterally directed process (Fig. 9–17) (much shorter non filamentous embolus, and median apophysis with two  processes in majority of *Araneus* s.s., e. g. *diadematus* group (Levi 1971)) and males have unmodified tibia II. *Araneus strandiellus* can be distinguished from sibling *Araneus pallasi* by having dorsal abdominal humps, and triangular scapus with pocket (wide, round scapus in *Araneus pallasi*). Males of these two species can be separated from other *Araneus* species by the round base of embolus, absence of the hump on tegulum and having longer median apophysis (Fig. 12, 16) with a triangular process in *Araneus strandiellus* (Fig. 14), and claw-like in *Araneus pallasi* (Fig. 15).

#### Description

(specimens from Kazakhstan)

#### .

Male. Total length 3.0. Carapace 1.4 long, 1.3 wide. Length of patella + tibia I 2.15 (patella 0.7; tibia 1.45). Carapace pale brown, covered with pale hairs; indistinctly darker on margins and with elongate whitish median spot (Fig. 6). Cephalic area of carapace slightly protruding. Diameter of AME subequal to PME. Distance between PME 1.3 times longer than between AME. Basal part of chelicera and retrolateral side dark brown. Promargin of chelicera with 3 teeth, retromargin with 2 small teeth. Sternum brown, with wide light spot in the centre (Fig. 7). Dorsum of abdomen with pair of small humps (Fig. 6). Humps separated by less than one diameter. Abdomen dark brown, with two white transverse bands. Venter of abdomen with dark median band, and whitish lateral bands (Fig. 7). Legs with annulations. Tibia II unmodified, similar to tibia I. Femur I prolaterally with 4 strong and long spines (Figs 5, 8) and with 7 short strong retrolateral spines.

Palp as in Figs 9–14. Patella with 2 macrosetae. Tegulum enlarged and all sclerites (embolus, conductor, radix, terminal and median apophyses) partly hidden by tegulum and cymbium. Terminal apophysis (*Ta*) long, flat, semicircular and weakly sclerotised; it runs apically between cymbium and tegulum. The long, thin and well sclerotised filiform embolus (*Em*) follows a groove in the terminal apophysis. Radix (*Ra*) short, stipes absent. Conductor (*Co*) very small, weakly sclerotised; supports tip of embolus from below. Median apophysis (*Ma*) with relatively small, triangular process (*Pm*) directed prolaterally.

**Figures 1–8.**
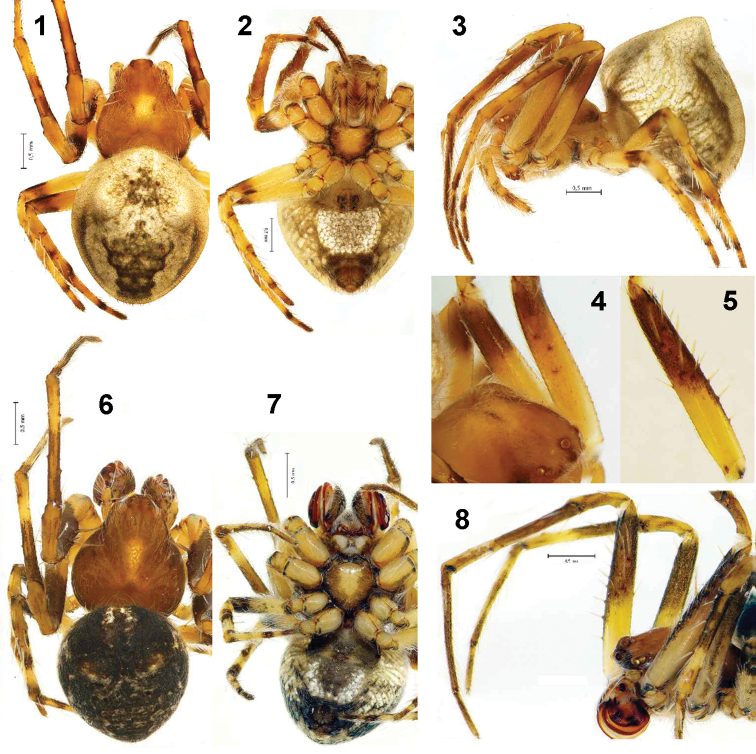
Habitus of *Araneus strandiellus*. **1–3** female, dorsal, ventral and lateral **4** female carapace and femora I and II, prolateral **5** male femur I, prolateral **6–7** male, dorsal and ventral **8** prosoma of male, lateral.

Female. Total length 2.75–4.0. Carapace 1.25–1.45 long, 1.2–1.4 wide. Length of patella + tibia I 1.9–2.15 (patella 0.6–0.7; tibia 1.3–1.5). Coloration and pattern of carapace as in male, but paler (Figs 1-3). Diameter of AME 1.3 times smaller than PME. Distance between PME 1.4 times longer than distance between AME. Cheliceral teeth as in male. Frontal part of chelicerae yellow, retrolateral side dark. White spot in the centre of sternum wider than in male.

Dorsum of abdomen with pair of conical humps separated by less than one diameter. Abdomen pale with dark pattern (Fig. 1). Venter of abdomen white between epigastric furrow and spinnerets; white area as wide as epigastric furrow (Fig. 2).

Femur I with 2–3 strong, long and pale spines (Figs 4). Legs yellow, with indistinct dark annulation. Ventral side of femur pale in almost all length. Patella pale with indistinct dark spot. Tibia and metatarsi without central dark rings or with small, dark spots.

Epigyne as in Figs 18–21, flat with weakly sclerotised inflexible triangular scapus (i.e. immovable merged with base of epigyne); tip of scapus with pocket (*Sp*); copulatory ducts and spermatheca slightly visible through cuticle. Base of epigyne always embedded in epigastric furrow, therefore posterior part visible only after its dissection or excavation.

**Figures 9–18.**
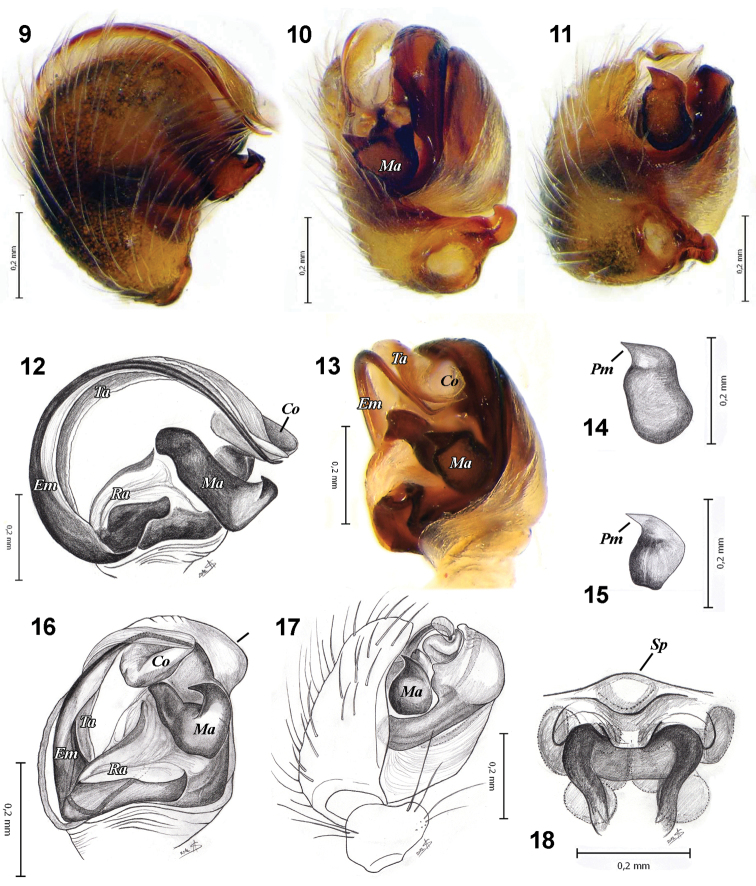
Copulatory organs of *Araneus strandiellus* (**9–14, 18**) and *Araneus pallasi* (**15–17**). **9–17** left male palp **9** prolateral **10** ventral-anterior **11** ventral-posterior **12** cymbium removed, prolateral **13** same, ventral **14** median apophysis, ventral **15** same **16** cymbium removed, prolateral **17** same, ventral **18** epigyne, posterior. ***Co*** conductor ***Em*** embolus ***Ma*** median apophysis ***Pm*** terminal process of median apophysis ***Ra*** radix ***Sp*** pocket on scapus of epigyne ***Ta*** terminal apophysis.

**Figures 19–21.**
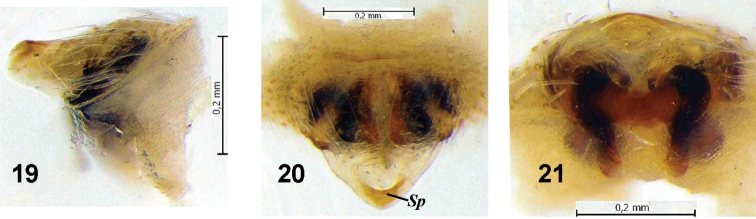
Epigyne of *Araneus strandiellus*. **19** lateral **20** ventral **21** posterior. ***Sp*** pocket on scapus of epigyne.

#### Variations.

Specimens from Tuva have darker coloration, lack white spot on carapace and sternum. Females from Tuva have no wide median band on the venter of abdomen. Importance of these differences is unclear to us.

#### Distribution.

**Figure 22.**
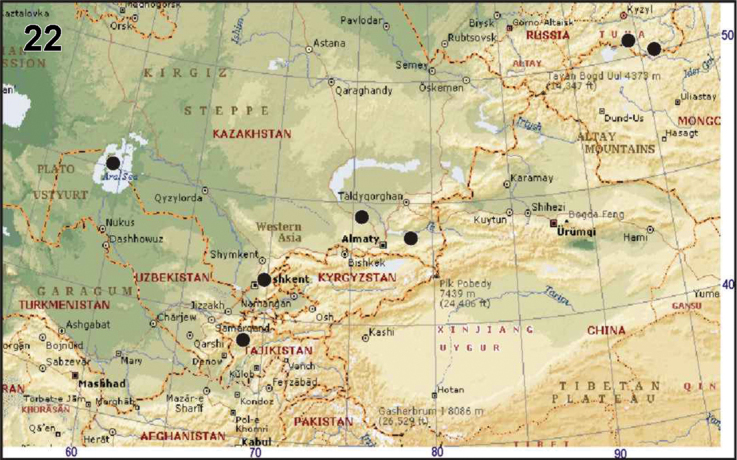
Known collecting localities of *Araneus strandiellus*.

The species is known from the Aral Sea to eastern Tuva (Fig. 22) south to Tajikistan.

#### Comments.

Generic affinity to *Araneus*,a genus comprising over 600 species (cf. Platnick 2012), is debatable. In comparison to *Araneus* s. s., *Araneus strandiellus* has only 3 promarginal and 2 retromarginal teeth (4 promarginal and 3 retromarginal in *Araneus* s.s.); females do not have heavy sclerotised epigyne and flexible scapus; and males lack stipes, subterminal apophysis and cap on embolus, conductor is very small and weak sclerotised and median apophysis has only one process.

Judging from the general shape of epigyne (presence of inflexible scapus) and the male palpal configuration (shape of median and terminal apophysis, embolus) *Araneus strandiellus* and probably the closest relative *Araneus pallasi* mostly resemble *Neoscona* Simon, 1864 (one of the junior synonyms of *Araneus pallasi* was considered in *Neoscona*) or *Agalenatea* Archer, 1951. However unlike *Araneus strandiellus* and *Araneus pallasi*, males of both *Agalenatea* and *Neoscona* have stipes and subterminal apophysis, an anticlockwise course of embolus, legs with hook on coxa I and modified tibia II (more numerous and stronger spines than on other legs). Epigyne of these two related species are weakly sclerotised and embedded in epigastric furrow (posterior part visible only after its dissection or excavation), while in *Agalenatea* and *Neoscona* epigyne are not embedded and heavy sclerotised.

## Supplementary Material

XML Treatment for Araneus strandiellus
